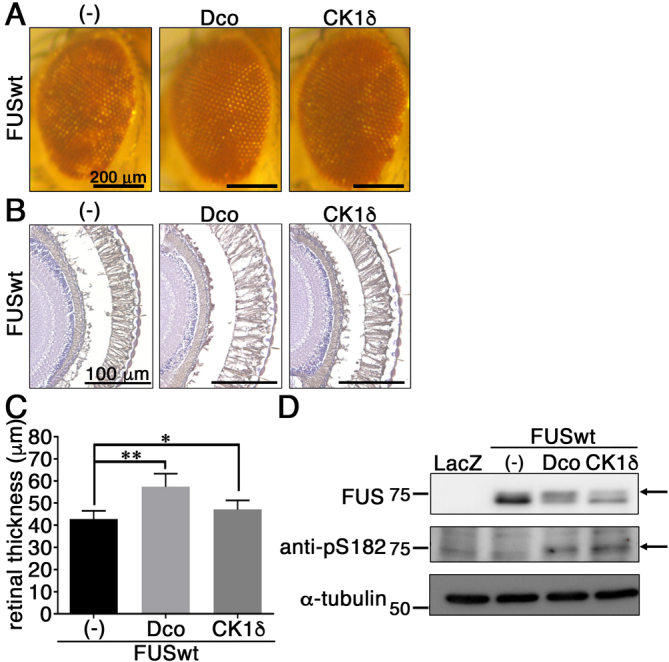# Casein kinase 1δ/ε phosphorylates fused in sarcoma (FUS) and ameliorates FUS-mediated neurodegeneration

**DOI:** 10.1016/j.jbc.2022.102191

**Published:** 2022-06-24

**Authors:** Yuya Kishino, Koji Matsukawa, Taisei Matsumoto, Ryota Miyazaki, Tomoko Wakabayashi, Takashi Nonaka, Fuyuki Kametani, Masato Hasegawa, Tadafumi Hashimoto, Takeshi Iwatsubo

**Affiliations:** 1 Department of Neuropathology, Graduate School of Medicine, The University of Tokyo, Tokyo, Japan; 2 Department of Pathology, Graduate School of Medicine, The University of Tokyo, Tokyo, Japan; 3 Department of Degenerative Neurological Diseases, National Institute of Neuroscience, National Center of Neurology and Psychiatry, Kodaira, Japan; 4 Department of Innovative Dementia Prevention, Graduate School of Medicine, The University of Tokyo, Tokyo, Japan; 5 Dementia Research Project, Tokyo Metropolitan Institute of Medical Science, Tokyo, Japan

**Keywords:** ALS, frontotemporal lobar degeneration, fused in sarcoma, CK1δ/ε, neurodegeneration, phosphorylation, *Drosophila*, cDNA, complementary DNA, CK1δ, casein kinase 1δ, DMSO, dimethyl sulfoxide, DNA-PK, DNA-dependent protein kinase, fALS, familial ALS, FTLD, frontotemporal lobar degeneration, FUS, fused in sarcoma, GAL4-UAS, GAL4-upstream activating sequence, GST, glutathione-*S*-transferase, HEK293, human embryonic kidney 293 cell line, LC, low-complexity, NP-40, Nonidet P-40, PY-NLS, proline–tyrosine nuclear localization signal, QGSY-rich, glutamine–glycine–serine–tyrosine rich, TDP-43, TAR DNA-binding protein 43, tg, transgenic

## Abstract

Aberrant cytoplasmic accumulation of an RNA-binding protein, fused in sarcoma (FUS), characterizes the neuropathology of subtypes of ALS and frontotemporal lobar degeneration, although the effects of post-translational modifications of FUS, especially phosphorylation, on its neurotoxicity have not been fully characterized. Here, we show that casein kinase 1δ (CK1δ) phosphorylates FUS at 10 serine/threonine residues *in vitro* using mass spectrometric analyses. We also show that phosphorylation by CK1δ or CK1ε significantly increased the solubility of FUS in human embryonic kidney 293 cells. In transgenic *Drosophila* that overexpress wt or P525L ALS-mutant human FUS in the retina or in neurons, we found coexpression of human CK1δ or its *Drosophila* isologue *Dco* in the photoreceptor neurons significantly ameliorated the observed retinal degeneration, and neuronal coexpression of human CK1δ extended fly life span. Taken together, our data suggest a novel regulatory mechanism of the assembly and toxicity of FUS through CK1δ/CK1ε-mediated phosphorylation, which could represent a potential therapeutic target in FUS proteinopathies.

ALS is a fatal neurodegenerative disorder characterized by the progressive loss of upper and lower motor neurons, resulting in severe atrophy of skeletal muscles. Most of the ALS cases are sporadic, but several missense mutations in the gene that encodes fused in sarcoma (FUS)/translated in liposarcoma have been identified in patients with autosomal dominant familial ALS (fALS) linked to fALS type 6 (1, 2, 3). Furthermore, aberrant cytoplasmic aggregates of FUS protein in degenerating neurons have been observed in the brains and spinal cords of patients with FUS-linked ALS (1, 2), frontotemporal lobar degeneration (FTLD) (4, 5), neuronal intermediate filament inclusion disease (6), and basophilic inclusion body disease (7), collectively referred to as FUS proteinopathies (8, 9).

FUS is an RNA-binding protein that belongs to the FET (*i.e.*, FUS, Ewing Sarcoma, TATA-binding protein–associated factor 15) protein family characterized by the presence of an RNA-recognition motif, a zinc finger domain, a nuclear export signal, and a proline–tyrosine nuclear localization signal (PY-NLS) (10). These domains play important roles in various aspects of RNA processing, for example, pre-mRNA splicing, long noncoding RNA expression, mRNA translation, and transport (11, 12). It remains unclear whether the loss of or gain of function of FUS is involved in the pathogenesis of FUS proteinopathies (13). ALS-linked mutations in the PY-NLS region disrupt the transportin-mediated nuclear import and lead to the cytoplasmic redistribution of FUS (14). Conditional overexpression of fALS-linked mutant FUS in the murine central nervous system caused motor neuron degeneration as well as structural and functional abnormalities at the neuromuscular junction (15). BAC transgenic (tg) mice of fALS-linked mutant FUS exhibited motor and cognitive dysfunctions with suppression of axonal protein synthesis (16). Furthermore, PY-NLS–deleted FUS knock-in mice displayed defects in the neuromuscular junction (17, 18). In contrast, FUS-deficient mice in the outbred background exhibited vacuolation in the neuropil of hippocampus, hyperactivity, and reduction in anxiety-like behavior, but no overt FTLD- or ALS-like phenotypes (19). These results support the hypothesis that toxic gain of function of FUS represents an important disease mechanism in FUS proteinopathies.

The amino-terminal region of FUS has been recognized as a low-complexity (LC) domain, that is, an intrinsically disordered region with amino acid composition primarily of serine, tyrosine, glycine, or glutamine. Biochemical studies have revealed that the LC domain of FUS is necessary and sufficient for hydrogel formation, liquid–liquid phase separation, and fibril formation (20, 21, 22, 23, 24, 25, 26, 27). Overexpression of the LC domain of FUS caused a reduction in *de novo* protein synthesis in cultured neurons (25). Overexpression of human FUS induced retinal or motor neuron degeneration in *Drosophila melanogaster*, whereas that of FUS lacking the QGSY-rich region or allS mutant FUS replaced at 27 tyrosine residues in the LC domain with serine, the latter being incapable of the self-assembly of FUS, completely abolished the toxicity (28, 29). These data strongly suggest that the LC domain of FUS is involved in the FUS accumulation and neurodegeneration in FUS proteinopathies (30).

DNA-dependent protein kinase (DNA-PK) is a serine/threonine protein kinase that is required for the nonhomologous end joining pathway of DNA repair. It has recently been reported that DNA-PK phosphorylates a set of serine/threonine residues in the LC domain of FUS and led to the cytoplasmic translocation of FUS in cells upon DNA damage (31, 32). Phosphorylation of FUS by DNA-PK has been shown to reduce the hydrogel formation or liquid–liquid phase separation of FUS (26, 33) and inhibit fibril formation (34) *in vitro*. These data support the notion that phosphorylation of the LC domain of FUS may be a mechanism by which cells govern the assembly of FUS in physiological and pathological conditions. However, it remains unclear whether phosphorylation of FUS by DNA-PK affects the neurodegeneration induced by FUS.

Casein kinase 1 (CK1) family kinases are serine/threonine–selective kinases that phosphorylate key regulatory molecules involved in Wnt signaling, NFκB signaling, and circadian rhythms (35, 36). CK1 family kinases have also been shown to phosphorylate a set of causative proteins for neurodegenerative disorders: CK1 phosphorylates tau protein (37) and disrupts the microtubule binding of tau *in vitro* (38), Ser129 of α-synuclein *in vitro* and in cells (39), and a set of serine/threonine residues within the carboxy-terminal glycine-rich region of TAR DNA-binding protein 43 (TDP-43) (40). It has also been shown that phosphorylation of TDP-43 by CK1δ triggers the cytoplasmic mislocalization and accumulation of TDP-43 (41). These previous results prompted us to speculate that the phosphorylation of causative proteins in neurodegenerative disorders by CK1 family kinases may affect the aberrant accumulation or aggregation of these proteins through structural changes. Here, we show that CK1δ and CK1ε phosphorylated a set of serine/threonine residues around the G-rich domain of FUS, corresponding to the latter half of the LC domain, *in vitro* and in human embryonic kidney 293 (HEK293) cells, and increased the solubility of FUS. Furthermore, overexpression of human or *Drosophila* ortholog of CK1δ in FUS tg flies ameliorated the FUS-mediated neuronal toxicity. Our results strongly support the notion that phosphorylation of the LC domain inhibits the assembly of FUS and FUS-mediated neurodegeneration through modification of the tertial structure of FUS.

## Results

### CK1δ and CK1ε phosphorylated serine/threonine residues of FUS *in vitro* and in HEK293 cells

**Figure 1:**
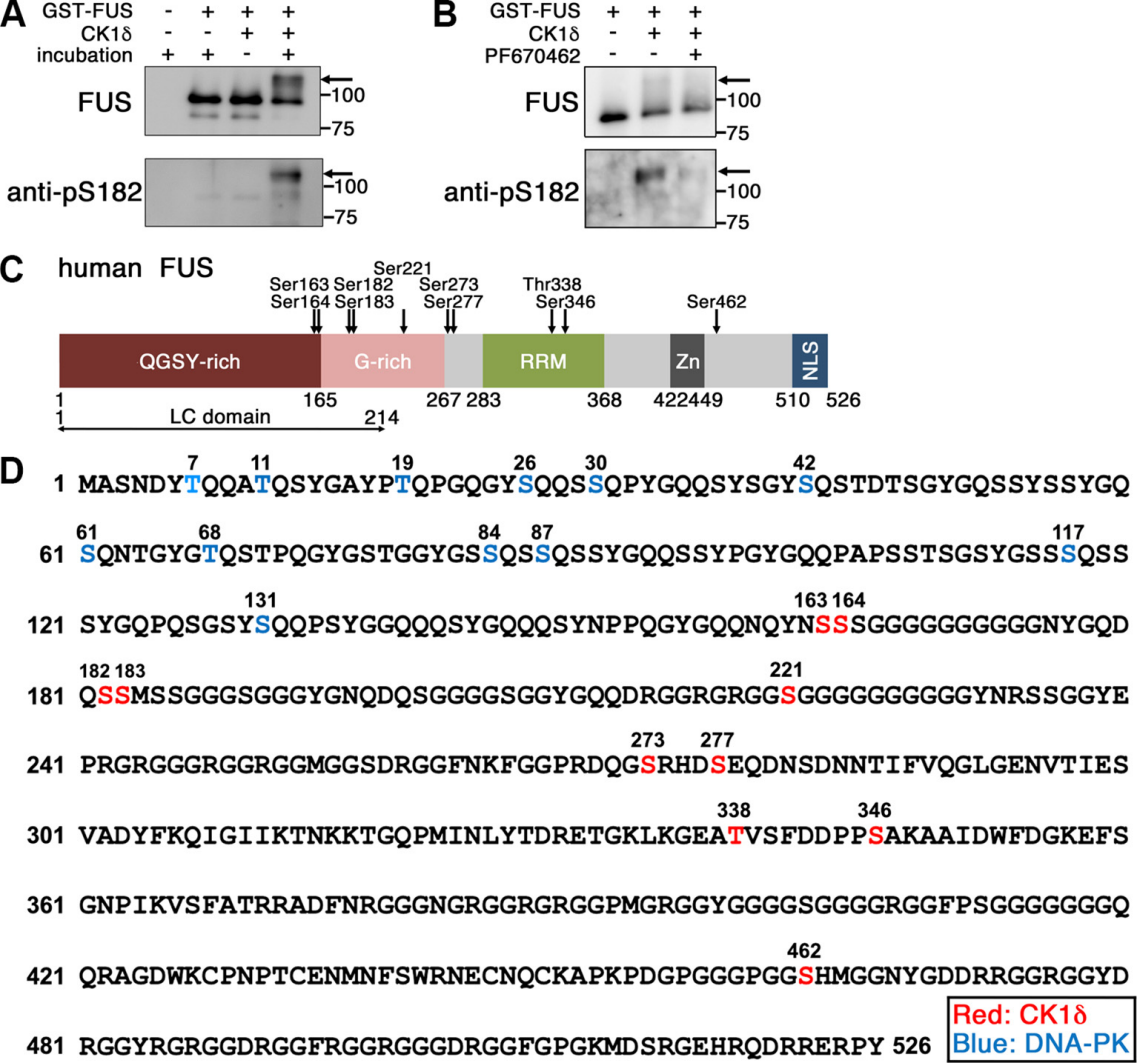
**Phosphorylation of FUS by CK1δ *in vitro*.***A*, immunoblot analyses of the products of *in vitro* kinase assay using an anti-FUS (*top panel*) or an anti-pS182 antibody (*bottom panel*). *B*, immunoblot analyses of the products of *in vitro* kinase assay with or without 10 μM of PF670462 with an anti-FUS (*top panel*) or an anti-pS182 antibody (*bottom panel*). *C*, schematic structure of human FUS protein. FUS possesses a QGSY-rich region, Gly-rich region, *low*-complexity (LC) domain, an RNA recognition motif (RRM), a zinc finger domain (Zn), and a proline–tyrosine nuclear localization signal (NLS). The location of the phosphorylated serine/threonine residues identified in this study is shown. *D*, CK1δ-phosphorylated sites on recombinant human FUS. Serine/threonine residues highlighted in *red* show the phosphorylated sites by CK1δ. Serine/threonine residues in the LC domain that fit with the DNA-PK-phosphorylated sites are highlighted in *blue*. CK1δ, casein kinase 1δ; DNA-PK, DNA-dependent protein kinase; FUS, fused in sarcoma; QGSY-rich, glutamine–glycine–serine–tyrosine rich.

To examine whether CK1 phosphorylates human FUS, glutathione-*S*-transferase (GST)-tagged human FUS (GST-FUS) purified from *Escherichia coli* was incubated with recombinant human CK1δ *in vitro* and separated by SDS-PAGE. GST-FUS was detected at a position of ∼90 kDa, which migrated slower at ∼110 kDa after incubation with CK1δ (Fig. 1*A*). Furthermore, the ∼110 kDa band was diminished by coincubation of CK1δ with alkaline phosphatase, or PF670462, a CK1δ and CK1ε selective kinase inhibitor (Fig. S1, *A* and *B*). These data suggest that FUS is phosphorylated by CK1δ *in vitro*. To further determine which serine/threonine residues of FUS were phosphorylated by CK1δ, we digested CK1δ-incubated GST-FUS in the gel with chymotrypsin and analyzed the digests by LC–MS/MS (40). Five phosphorylated polypeptides were obtained, in which 10 phosphorylated sites of FUS, that is, Ser163, Ser164, Ser182, Ser183, Ser221, Ser273, Ser277, Thr338, Ser346, and Ser462 were identified, which were distinct from those reported to be phosphorylated by DNA-PK (Figs. 1, *C*, *D*, S2 and Table S1) (31, 32, 33). Among the 10 serine/threonine residues, seven (*i.e.*, Ser163, Ser164, Ser182, Ser183, Ser221, Ser273, and Ser277) were located within or in the vicinity of the G-rich domain of FUS; interestingly, Ser182 and Ser183 residues are located at the C-terminal end of the LC region of FUS (Fig. 1*C*), whereas TDP-43 is phosphorylated at Ser409 and Ser410 residues by CK1δ, which are located at the end of the glycine-rich LC region (41). This led us to generate polyclonal phosphospecific antibodies against FUS phosphorylated at Ser182 (anti-pS182) or simultaneously at Ser182 and Ser183 (anti-pS182/pS183) and found that the ∼110 kDa GST-FUS on SDS-PAGE was positively labeled either by anti-pS182 (Fig. 1, *A*, and *B*) or anti-pS182/pS183 (Fig. S1*B*).

**Figure 2:**
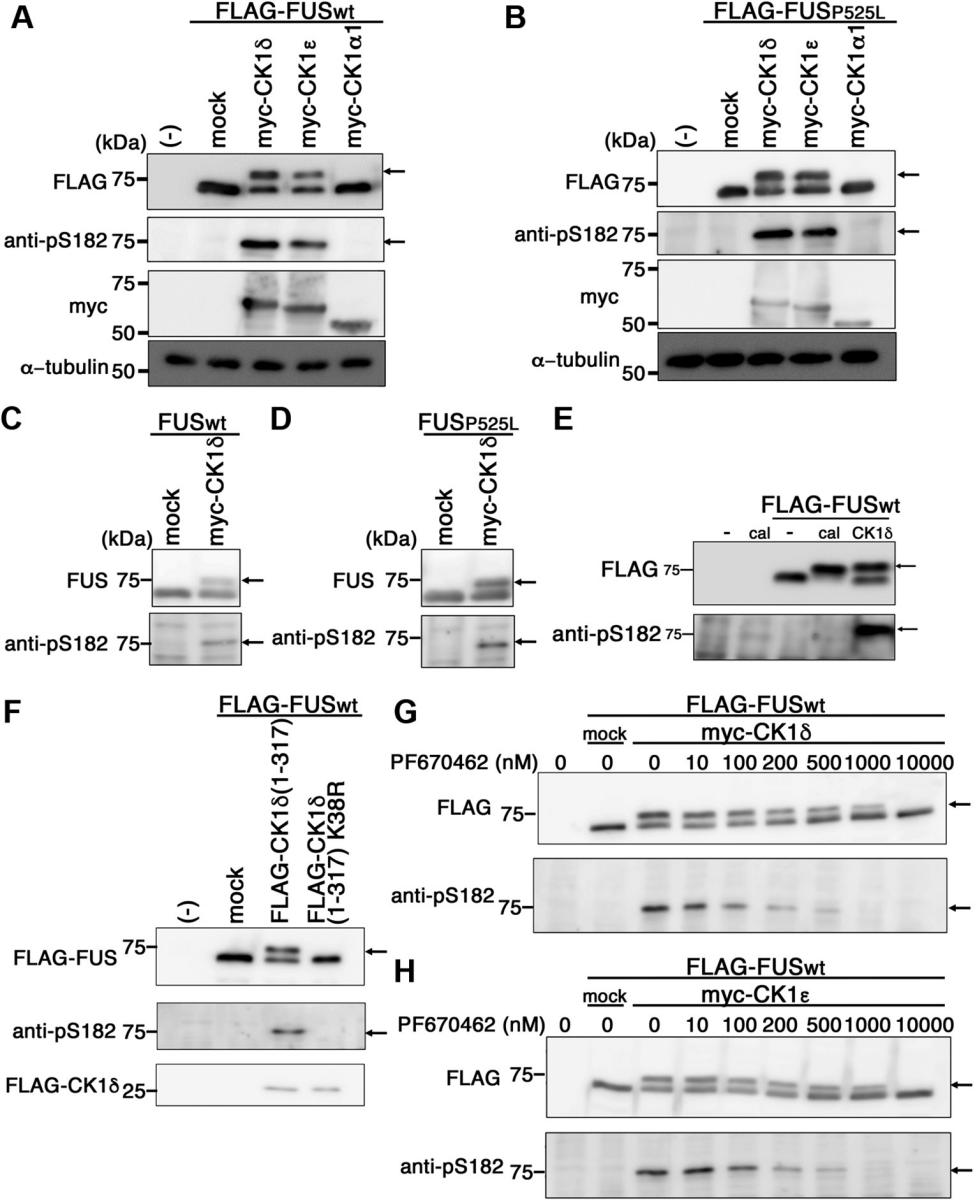
**Expression of CK1δ or CK1ε caused FUS phosphorylation in HEK293 cells.***A* and *B*, immunoblot analyses of the lysates of HEK293 cells expressing FLAG-tagged human wt FUS (*A*) or FLAG-tagged P525L mutant FUS (*B*) with CK1δ, CK1ε, or CK1α1, by an anti-FLAG antibody (*top panel*), an anti-pS182 antibody (*second upper panel*), an anti-myc antibody (*third upper panel*), or an anti-α-tubulin antibody (*bottom panel*). *C* and *D*, immunoblot analyses of the lysates of HEK293 cells expressing wt FUS (*C*) or P525L mutant FUS (*D*) with CK1δ or mock transfection by an anti-FUS antibody (*top panel*) or an anti-pS182 antibody (*bottom panel*). *E*, immunoblot analyses of the lysates of HEK293 cells treated with 20 nM of calicheamicin (cal), cells expressing FLAG-tagged wt FUS, FLAG-tagged wt FUS treated with 20 nM of cal, or HEK293 cells expressing FLAG-tagged wt FUS and CK1δ by an anti-FLAG antibody (*top panel*), or an anti-pS182 antibody (*bottom panel*). *F*, immunoblot analyses of the lysates of HEK293 cells expressing FLAG-tagged human wt FUS with CK1δ (1–317), or CK1δ (1–317) K38R, by an anti-FLAG antibody for FUS (*top panel*) or for CK1δ (*bottom panel*), or an anti-pS182 antibody (*middle panel*). *G* and *H*, immunoblot analyses of the lysates of HEK293 cells expressing FLAG-tagged human wt FUS with CK1δ (*G*) or CK1ε (*H*) in the presence of PF670462 at concentrations of 0, 10, 100, 200, 500, 100, and 10,000 nM by an anti-FLAG antibody (*top panel*) or an anti-pS182 antibody (*bottom panel*). *Arrows* indicate bands that correspond to FUS polypeptide phosphorylated by CK1δ. CK1, casein kinase 1; FUS, fused in sarcoma; HEK293, human embryonic kidney 293 cell line.

We next examined whether human FUS is phosphorylated by the CK1 family kinases in mammalian cells. We cotransfected FLAG-tagged wt human FUS with myc-tagged CK1α1, CK1δ, or CK1ε in HEK293 cells and separated them by SDS-PAGE. Immunoblot analysis showed that wt FUS polypeptides cotransfected with CK1δ or CK1ε were detected as ∼80 and 70 kDa bands, whereas wt FUS cotransfected with CK1α1 or mock transfected was detected at ∼70 kDa (Fig. 2*A*). Immunoblot analysis with anti-pS182 exclusively labeled the 80 kDa band, which suggested that human wt FUS is phosphorylated by CK1δ or CK1ε but not by CK1α1. fALS-linked P525L mutant FUS was also phosphorylated by CK1δ or CK1ε in HEK293 cells (Fig. 2*B*). To rule out the possibility that FLAG tag affected the phosphorylation of FUS by CK1 δ/ε, we cotransfected nontagged wt or P525L mutant FUS with myc-tagged CK1δ in HEK293 cells and found that Ser182 residue of nontagged wt or P525L mutant FUS was phosphorylated by CK1δ as in FLAG-tagged FUS (Fig. 2, *C* and *D*).

To confirm the specificity of the anti-pS182 antibody, we replaced the Ser182 or Ser182/Ser183 residues of FUS with alanine (S182A and S182A/S183A, respectively) and cotransfected FLAG-tagged wt, S182A or S182A/S183A FUS with CK1δ in HEK293 cells. S182A or S182A/S183A FUS polypeptides were migrated both at ∼80 and 70 kDa in a similar manner to wt FUS; however, these bands were hardly detected by anti-pS182 (Fig. S1*C*). Furthermore, the preabsorption of the anti-pS182 antibody with a phosphorylated polypeptide (GNYGQDQ(pS)SMSSGGG, corresponding to the sequence 175–189 of FUS with phosphoserine182), but not with a nonphosphorylated polypeptide, abolished the detection of the ∼80 kDa band in the lysate of HEK293 cells doubly transfected with FLAG-tagged FUSwt and myc-tagged CK1δ (Fig. S1*D*). These data suggest that anti-pS182 specifically recognized FUS phosphorylated at Ser182. To further examine whether DNA-PK phosphorylates Ser182 of FUS in HEK293 cells, we incubated HEK293 cells transfected with FLAG-FUSwt with 20 nM of calicheamicin (MedChemExpress), an inducer of DNA double-strand breaks, to elicit the activation of DNA-PK; however, no bands were detected by anti-pS182 FUS, despite the retarded mobility of the FLAG-positive band indicative of FUS phosphorylation by DNA-PK (Fig. 2*E*), suggesting that DNA-PK does not phosphorylate Ser182 of FUS.

To further examine whether the kinase activity of CK1δ or CK1ε is necessary for the phosphorylation of FUS, we transfected HEK293 cells with FLAG-tagged FUSwt with FLAG-tagged CK1δ1-317, a hyperactive form of CK1δ, or FLAG-tagged CK1δ1-317 K38R, a kinase-dead mutant form of CK1δ (41) and found that CK1δ1-317 phosphorylated FUS exhibiting an additional band with slower migration, similarly to CK1δ (Fig. 2*A*), whereas FUS was not phosphorylated by CK1δ1-317 K38R (Fig. 2*F*). In addition, we cotransfected HEK293 cells with FLAG-tagged FUSwt and either myc-tagged CK1δ or CK1ε, in the presence of different concentrations of PF670462 ranging from 10 to 10,000 nM, and observed a dose-dependent inhibition of CK1δ- or CK1ε-induced FUS phosphorylation by PF670462 (Fig. 2, *G* and *H*). These data strongly suggest that FUS is phosphorylated by CK1δ or CK1ε in a kinase activity–dependent manner.

### Phosphorylation by CK1δ or CK1ε resulted in an increase in the solubility of FUS

**Figure 3:**
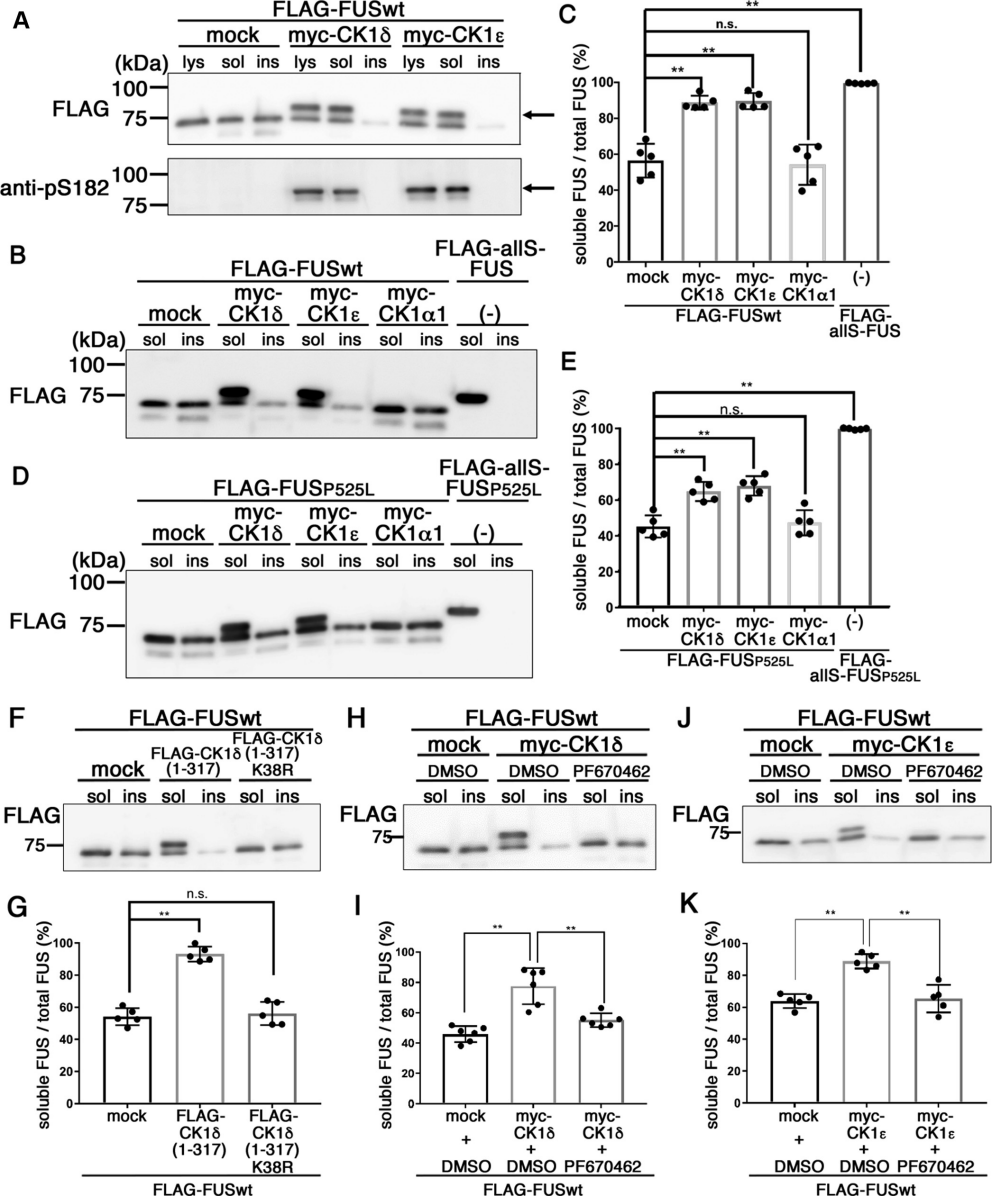
**Increased solubility of FUS phosphorylated by CK1δ or CK1ε in HEK293 cells.** Lysates were separated into NP-40 soluble (sol) and insoluble fractions (ins). *A*, immunoblot analyses of the samples of HEK293 cells expressing FLAG-tagged wt FUS and CK1δ or CK1ε with an anti-FLAG (*top panel*) or an anti-pS182 (*bottom panel*) antibody. “lys” represents the lysate of HEK293 cells lysed in 8 M urea buffer without fractionation. *Arrows* indicate the bands corresponding to the phosphorylated FUS. *B* and *D*, immunoblot analyses of the samples of HEK293 cells expressing FLAG-tagged wt FUS (*B*) or FLAG-tagged P525L mutant FUS (*D*) with an anti-FLAG antibody (*top panel*) or an anti-pS182 antibody (*bottom panel*). *C* and *E*, quantification of the solubility of FLAG-tagged wt FUS (*C*) or FLAG-tagged P525L mutant FUS (*E*). One-way ANOVA with Dunnett’s test. *F*, immunoblot analyses of the samples of HEK293 cells expressing FLAG-tagged wt FUS and CK1δ (1–317) or CK1δ (1–317) K38R by an anti-FLAG antibody. *G*, quantification of the solubility of FLAG-tagged wt FUS in (*F*). One-way ANOVA with Dunnett’s test (n = 5). *H* and *J*, immunoblot analyses of the samples of HEK293 cells expressing FLAG-tagged wt FUS and CK1δ (*H*) or CK1ε (*J*) with 1 μM of PF670462 by an anti-FLAG antibody. *I* and *K*, quantification of the solubility of FLAG-tagged wt FUS in (*H*) and (*J*), respectively. Error bars show SD. One-way ANOVA with Dunnett’s test (n = 6 in *I* and n = 5 in *K*). ∗∗*p* < 0.01. CK1, casein kinase 1; FUS, fused in sarcoma; HEK293, human embryonic kidney 293 cell line; NP-40, Nonidet P-40.

It has been reported that protein extracts from postmortem FTLD-FUS patients exhibited an increase in the insolubility of FUS (42). Conversely, we have reported that allS mutation of FUS, in which 27 tyrosine residues within the LC domain were replaced with serine, caused reduction in its self-assembly and increased the solubility of FUS on wt or P525L mutant basis (Fig. 3, *B*–*E*) (28). These observations led us to examine the effect of phosphorylation of FUS by CK1δ/ε on its solubility. We cotransfected FLAG-tagged wt FUS with myc-tagged CK1δ or CK1ε in HEK293 cells and lysed the cells with 1% Nonidet P-40 (NP-40) buffer to extract the detergent-soluble fraction. After centrifugation, the pellet was resolubilized by 8 M urea–3% SDS buffer to obtain the detergent-insoluble fraction (28). We found that a major proportion of FUS proteins cotransfected with CK1δ or CK1ε were extracted into the detergent-soluble fraction, whereas FUS proteins with mock transfection were distributed both in detergent-soluble and detergent-insoluble fractions (Fig. 3*A*). Notably, the ∼80 kDa pS182-phosphorylated FUS polypeptide was hardly detected in the detergent-insoluble fraction (Fig. 3*A*). The ratios of soluble FUS relative to total FUS were significantly higher upon cotransfection with CK1δ or CK1ε compared with those with CK1α1 or mock transfection (56.9 ± 8.5% in mock, 89.8 ± 4.2% in CK1δ, 90.5 ± 4.6% in CK1ε, 55.1 ± 10.3% in CK1α1, and 99.5 ± 0.2% in allS-wt FUS; Fig. 3, *B* and *C*). We also examined the solubility of P525L ALS mutant FUS phosphorylated by CK1 family kinases and found that the ratios of soluble P525L mutant FUS cotransfected with CK1δ or CK1ε also were significantly higher than those with CK1α1 or mock transfection (44.6 ± 6.2% in mock, 64.1 ± 5.3% in CK1δ, 67.2 ± 5.4% in CK1ε, 46.7 ± 7.1% in CK1α1, and 98.9 ± 0.4% in allS-P525L mutant FUS; Fig. 3, *D* and *E*). These data altogether show that phosphorylation of FUS by CK1δ or CK1ε increases the solubility of FUS.

To examine whether the kinase activity of CK1δ or CK1ε is necessary for the increase in the solubility of FUS, we cotransfected HEK293 cells with FLAG-tagged FUSwt with FLAG-tagged CK1δ1-317 or FLAG-tagged CK1δ1-317 K38R. The ratio of soluble FUS relative to total FUS was significantly increased upon cotransfection with CK1δ1-317 compared with that with mock transfection, whereas they were comparable between CK1δ1-317 K38R and mock transfection (54.2 ± 5.3% in mock, 93.1 ± 4.6% in CK1δ1-317, and 56.1 ± 7.2% in CK1δ1-317 K38R; Fig. 3, *F* and *G*). We further cotransfected HEK293 cells with FLAG-tagged FUSwt and either myc-tagged CK1δ or CK1ε in the presence of 1 μM of PF670462 and found that PF670462 suppressed the increase in the solubility of FUS by CK1δ (46.0 ± 5.3% in mock + dimethyl sulfoxide [DMSO], 77.6 ± 12.0% in CK1δ + DMSO, and 55.1 ± 4.5% in CK1δ + PF670462; Fig. 3,*H* and *I*) or CK1ε (63.8 ± 4.4% in mock + DMSO, 88.8 ± 4.6% in CK1ε + DMSO, and 65.3 ± 8.6% in CK1ε + PF670462; Fig. 3, *J* and *K*). These data suggest that phosphorylation of FUS by CK1δ or CK1ε increased the solubility of FUS in a kinase activity–dependent manner.

### Phosphorylation by CK1δ or CK1ε did not alter the subcellular distribution of FUS

**Figure 4:**
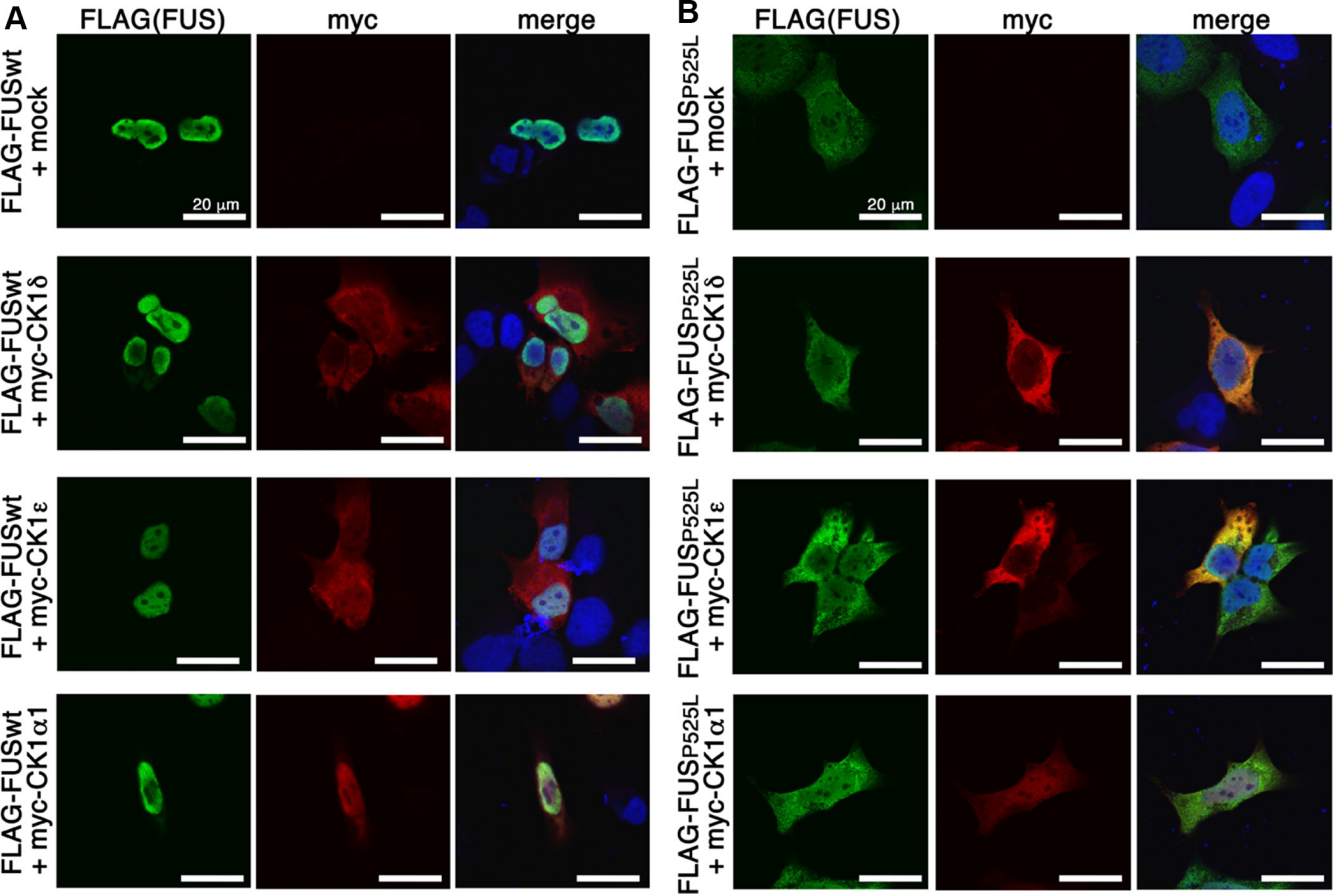
**Expression of CK1δ or CK1ε did not alter the subcellular distribution of FUS in HEK293 cells.***A* and *B*, immunofluorescence labeling of HEK293 cells transfected with FLAG-tagged wt FUS (*A*) or FLAG-tagged P525L mutant FUS (*B*) with mock (*upper panels*), myc-tagged CK1δ (*second upper panels*), myc-tagged CK1ε (*third upper panels*), or myc-tagged CK1α1 (*bottom panels*) by an anti-FLAG antibody (*green*), an anti-myc antibody (*red*), or DRAQ5 (*blue*) as a marker for cell nucleus. The scale bars represent 20 μm. CK1, casein kinase 1; FUS, fused in sarcoma; HEK293, human embryonic kidney 293 cell line.

FUS is mainly localized in the nucleus, whereas P525L mutant FUS is localized to the cytoplasm because of the disruption of nuclear import mediated by transportin (14). It has been reported that phosphorylation by DNA-PK mediated cytoplasmic translocation of FUS in HEK293T cells (32). To investigate whether phosphorylation by CK1δ or CK1ε alters the subcellular localization of FUS, we cotransfected HEK293 and human neuroblastoma SH-SY5Y cells with FLAG-tagged FUSwt or FUS P525L, together with myc-tagged CK1δ, CK1ε, or CK1α1. Immunoblot analysis revealed that CK1δ and CK1ε phosphorylated FUSwt or FUS P525L in SH-SY5Y cells, but CK1α1 did not (Fig. S3*A*). Immunofluorescence labeling showed that FUSwt cotransfected with CK1δ or CK1ε was mainly localized to the nucleus, similarly to the results of cotransfection with CK1α1 or mock transfection either in HEK293 or SH-SY5Y cells (Figs. 4*A*, and S3*B*). We also found that P525L mutant FUS cotransfected with CK1δ or CK1ε was mainly localized to the cytoplasm, which was similar to the results upon cotransfection with CK1α1 or mock transfection, either in HEK293 or SH-SY5Y cells (Figs. 4*B*, and S3*C*). These data suggest that phosphorylation of FUS by CK1δ or CK1ε does not alter its subcellular localization in HEK293 or SH-SY5Y cells.

### Overexpression of CK1δ ameliorated the FUS-mediated retinal degeneration

**Figure 5:**
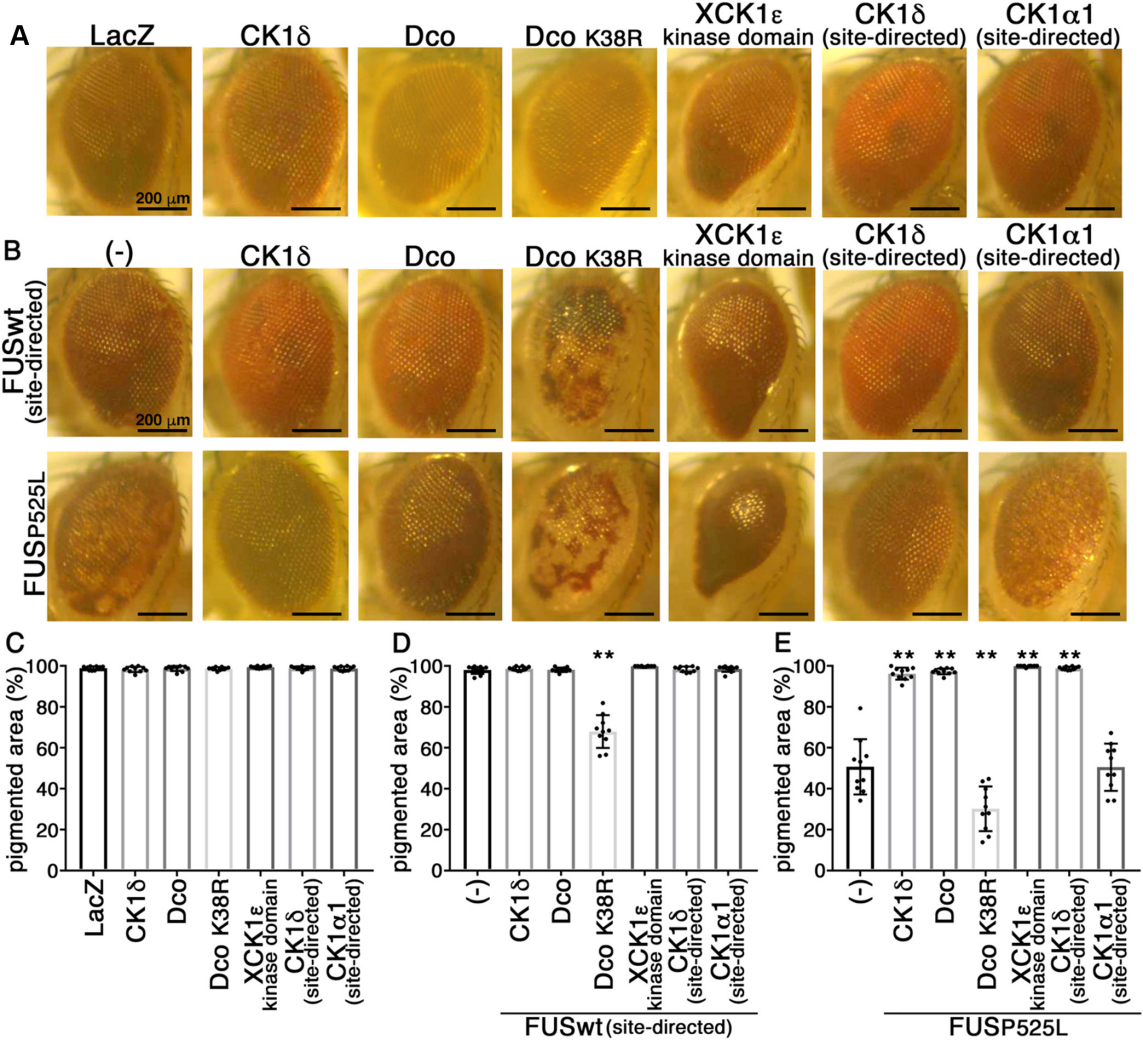
**Expression of CK1δ, Dco, or *Xenopus* CK1ε ameliorated the eye degeneration phenotypes of FUS wt or P525L tg flies.***A*, external pictures of eyes of 20-day-old tg flies expressing LacZ, CK1δ, Dco, Dco K38R, XCK1ε kinase domain, CK1δ (site-directed), or CK1α1 (site-directed). The scale bars represent 200 μm. *B*, external pictures of eyes of 20-day-old tg flies coexpressing CK1δ, Dco, Dco K38R, XCK1ε kinase domain, CK1δ (site-directed), or CK1α1 (site-directed) with FUSwt (site-directed) (*upper panels*) or FUS P525L (*lower panels*). The scale bars represent 200 μm. *C*–*E*, quantification of pigmented area (%) of tg flies singly expressing LacZ, CK1δ, Dco, Dco K38R, XCK1ε kinase domain, CK1δ (site-directed), or CK1α1 (site-directed) (*C*), doubly expressing FUSwt (site-directed) with CK1δ, Dco, Dco K38R, XCK1ε kinase domain, CK1δ (site-directed), or CK1α1 (site-directed) (*D*), or double expressing of FUS P525L with CK1δ, Dco, Dco K38R, XCK1ε kinase domain, CK1δ (site-directed), or CK1α1 (site-directed) (*E*). Mean ± SD. One-way ANOVA with Dunnett’s test (n = 10). ∗∗*p* < 0.01. CK1, casein kinase 1; FUS, fused in sarcoma; tg, transgenic.

To investigate into the effect of phosphorylation of FUS by CK1δ or CK1ε on FUS-mediated neurodegeneration, we utilized tg *D. melanogaster*–overexpressing human wt or P525L ALS-linked mutant FUS (FUS wt or P525L tg flies, respectively) we previously established using a *GAL4-UAS* system, which exhibit neurodegeneration in the retinal photoreceptor neurons (28). To avoid the effects of transgene insertion on the expression of gene(s) nearby the integration site, we also generated site-directed insertion line of human wt FUS (FUSwt [site-directed]) using an attB-attP40 site-directed insertion technique. We generated double tg flies that overexpress either *UAS-human CK1δ*, *UAS-discs overgrown/doubletime* (Dco, a *Drosophila* homolog of CK1δ/ε) (43), *UAS-Dco K38R* (kinase-dead mutant of Dco), *UAS-Xenopus CK1ε kinase-domain* (XCK1ε, 97% identical to human CK1ε) (44, 45), site-directed insertion line of *UAS-human CK1δ* (CK1δ [site-directed]), or site-directed insertion line of *UAS-human CK1α1* (CK1α1 [site-directed]), and either FUSwt (site-directed) or FUS P525, and crossed them with *gmr-GAL4* flies to drive the expression of transgenes in retinal cells. The external surface of the eyes of 20-day-old FUS P525L single tg flies exhibited more severe degenerative phenotype, that is, loss of pigment or necrotic patches in the eye, compared with those of FUSwt (site-directed) single tg flies (Fig. 5*B*). Notably, coexpression of CK1δ, Dco, XCK1ε kinase domain, or CK1δ (site-directed) in the retina of FUS P525L tg flies significantly alleviated the degenerative phenotypes compared with those in FUS P525L single tg flies, whereas coexpression of Dco K38R or CK1α1 (site-directed) did not (pigmented area: 50.7 ± 13.5% in FUS P525L single, 96.2 ± 2.9% in FUS P525L + CK1δ, 97.3 ± 1.4% in FUS P525L + Dco, 30.2 ± 10.9% in FUS P525L + Dco K38R, 99.9 ± 0.3% in FUS P525L + XCK1ε kinase domain, 98.8 ± 0.8% in FUS P525L + CK1δ [site-directed], 50.5 ± 11.5 in FUS P525L + CK1α1 [site-directed]; Fig. 5, *B* and *E*). The eyes of 20-day-old tg flies that singly express LacZ, CK1δ, Dco, Dco K38R, XCK1ε kinase domain, CK1δ (site-directed), or CK1α1 (site-directed) exhibited no such degenerative phenotypes (pigmented area: 98.9 ± 0.9% in LacZ, 98.3 ± 1.3% in CK1δ, 98.7 ± 1.3% in Dco, 98.6 ± 0.7% in Dco K38R, 99.3 ± 0.6% in XCK1ε kinase domain, 98.9 ± 0.9% in CK1δ [site-directed], 98.7 ± 1.0% in CK1α1 [site-directed]; Fig. 5, *A* and *C*). The eyes of 20-day-old tg flies that coexpress FUSwt (site-directed) with CK1δ, Dco, XCK1ε kinase domain, CK1δ (site-directed), or CK1α1 (site-directed) did not exhibit degenerative phenotypes too (pigmented area: 98.1 ± 1.9% in FUSwt single, 98.7 ± 0.9% in FUSwt + CK1δ, 98.1 ± 1.1% in FUSwt + Dco, 99.9 ± 0.2% in FUSwt + XCK1ε kinase domain, 98.4 ± 1.3% in FUSwt + CK1δ [site-directed], 98.6 ± 1.5% in FUSwt + CK1α1 [site-directed]; Fig. 5, *B* and *D*). These data suggest that overexpression of CK1δ or CK1ε alleviated the retinal degeneration induced by FUS P525L in a kinase activity–dependent manner. The 20-day-old tg flies coexpressing Dco K38R with FUSwt (site-directed) or FUS P525L exhibited more severe degenerative phenotypes in the eye compared with FUSwt (site-directed) or FUS P525L single tg flies, respectively (pigmented area: 67.9 ± 8.0% in FUSwt + Dco K38R, 30.2 ± 10.9% in FUS P525L + Dco K38R; Fig. 5, *B*, *D* and *E*). Dco is essential for the development of eye or wing *via* Wingless signaling (43), indicating that Dco K38R may inhibit the intrinsic Dco activity in a dominant-negative manner (46), leading to the degenerative phenotypes in the eye, although we cannot exclude the possibility that overexpression of Dco K38R induced the toxicity independent of Dco kinase activity.

**Figure 6:**
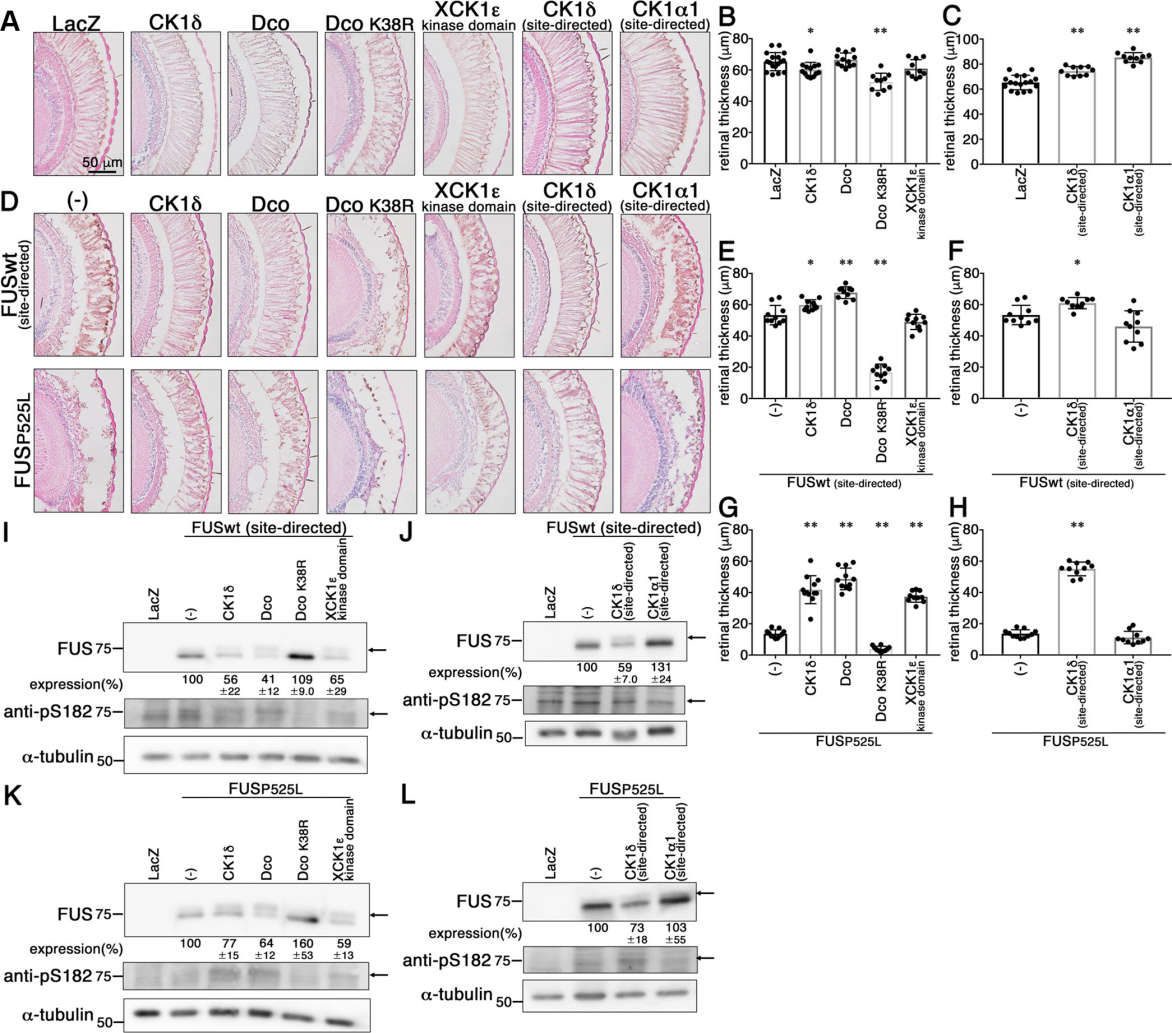
**Expression of CK1δ, Dco, or *Xenopus* CK1ε ameliorated the retinal degeneration of FUS wt or P525L mutant tg flies.***A*, hematoxylin–eosin stained sections of eyes of 5-day-old tg flies expressing LacZ, CK1δ, Dco, Dco K38R, XCK1ε kinase domain, CK1δ (site-directed), or CK1α1 (site-directed). The scale bar represents 100 μm. *B* and *C*, quantification of the retinal thickness in tg flies singly expressing LacZ, CK1δ, Dco, Dco K38R, XCK1ε kinase domain, CK1δ (site-directed), or CK1α1 (site-directed). Mean ± SD. One-way ANOVA with Dunnett’s test (n = 17 in LacZ, n = 13 in CK1δ, n = 12 in Dco, n = 10 in Dco K38R, n = 10 in XCK1ε kinase domain, n = 10 CK1δ [site-directed], and n = 10 CK1α1 [site-directed]). ∗*p* < 0.05, ∗∗*p* < 0.01. *D*, hematoxylin–eosin stained sections of eyes of 10-day-old tg flies coexpressing CK1δ, Dco, Dco K38R, XCK1ε kinase domain, CK1δ (site-directed), or CK1α1 (site-directed) with FUSwt (site-directed) (*upper panels*) or 5-day-old tg flies coexpressing CK1δ, Dco, Dco K38R, XCK1ε kinase domain, CK1δ (site-directed), or CK1α1 (site-directed) with FUS P525L (*lower panels*). *E* and *F*, quantification of retinal thickness in tg flies doubly expressing FUSwt (site-directed) with CK1δ, Dco, Dco K38R, XCK1ε kinase domain, CK1δ (site-directed), or CK1α1 (site-directed). Mean ± SD. One-way ANOVA with Dunnett’s test (n = 10). ∗*p* < 0.05, ∗∗*p* < 0.01. *G* and *H*, quantification of the retinal thickness in tg flies doubly expressing FUS P525L with CK1δ, Dco, Dco K38R, XCK1ε kinase domain, CK1δ (site-directed), or CK1α1 (site-directed). Mean ± SD. One-way ANOVA with Dunnett’s test (n = 11 in FUS P525L, n = 12 in CK1δ, n = 11 in Dco, n = 10 in Dco K38R, n = 10 in XCK1ε kinase domain, n = 10 CK1δ [site-directed], n = 10 CK1α1 [site-directed]). ∗∗*p* < 0.01. *I* and *J*, immunoblot analyses of the heads of 1-day-old tg flies with an anti-FUS antibody (*top panel*), an anti-pS182 antibody (*second upper panel*), or an anti-α-tubulin antibody (*bottom panel*) in LacZ, FUSwt (site-directed) single tg flies, FUSwt (site-directed) and CK1δ, Dco, Dco K38R, XCK1ε kinase domain, CK1δ (site-directed), or CK1α1 (site-directed) double tg flies. Relative expression levels of FUSwt are indicated under the *top panels* (n = 3). *K* and *L*, immunoblot analyses of the heads of 1-day-old tg flies with an anti-FUS antibody (*top panel*), an anti-pS182 antibody (*second upper panel*), or an anti-α-tubulin antibody (*bottom panel*) in LacZ, FUS P525L single tg flies, FUS P525L and CK1δ, Dco, Dco K38R, XCK1ε kinase domain, CK1δ (site-directed), or CK1α1 (site-directed) double tg flies. Relative expression levels of FUS P525L are indicated under the *top panels* (n = 3 in *K* and n = 5 in *L*). CK1, casein kinase 1; FUS, fused in sarcoma; tg, transgenic.

Histologically, overexpression of CK1δ or Dco K38R in the retina caused mild but significant reduction in the thickness of the retina compared with that in LacZ tg flies, whereas overexpression of Dco, XCK1ε kinase domain, CK1δ (site-directed), or CK1α1 (site-directed) never elicited retinal degeneration (retinal thickness [μm]: 65.3 ± 5.8 in LacZ, 60.2 ± 4.7 in CK1δ, 66.6 ± 4.3 in Dco, 52.4 ± 5.5 in Dco K38R, 60.9 ± 5.7 in XCK1ε kinase domain, 74.5 ± 3.6 in CK1δ [site-directed], 85.3 ± 3.9 in CK1α1 [site-directed]; Fig. 6, *A*–*C*). Remarkably, overexpression of Dco, CK1δ, or CK1δ (site-directed) significantly ameliorated the FUS-mediated retinal degeneration, that is, vacuolation and thinning of the thickness, in the retina of 10-day-old FUS wt (site-directed) tg flies (retinal thickness [μm]: 53.4 ± 6.2 in FUSwt single, 59.9 ± 3.3 in FUSwt + CK1δ, 67.8 ± 3.8 in FUSwt + Dco, 16.8 ± 5.3 in FUSwt + Dco K38R, 49.0 ± 4.9 in FUSwt + XCK1ε kinase domain, 61.0 ± 3.6 in FUSwt + CK1δ [site-directed], 46.1 ± 10.0 in FUSwt + CK1α1 [site-directed]; Fig. 6, *D*–*F*), and overexpression of Dco, CK1δ, XCK1ε kinase domain, or CK1δ (site-directed) dramatically ameliorated the FUS-mediated retinal degeneration in the retina of 5-day-old FUS P525L tg flies (retinal thickness [μm]: 13.6 ± 2.5 in FUS P525L single, 41.8 ± 8.9 in FUS P525L + CK1δ, 48.8 ± 6.9 in FUS P525L + Dco, 4.0 ± 1.7 in FUS P525L + Dco K38R, 37.4 ± 3.5 in FUS P525L + XCK1ε kinase domain, 55.1 ± 4.3 in FUS P525L + CK1δ [site-directed], 11.2 ± 4.0 in FUS P525L + CK1α1 [site-directed]; Fig. 6, *D*, *G*, and *H*). Similarly to FUSwt (site-directed) tg flies, overexpression of Dco or CK1δ significantly ameliorated the retinal degeneration in the retina of 5-day-old FUS wt tg flies (Fig. S4).

Immunoblot analyses of the lysates of heads of FUS tg flies showed that FUS proteins expressed in the retina of 1-day-old double tg flies (*i.e.*, FUSwt [site-directed]/CK1δ, FUSwt [site-directed]/Dco, FUSwt [site-directed]/XCK1ε kinase domain, FUSwt [site-directed]/CK1δ [site-directed], FUS P525L/CK1δ, FUS P525L/Dco, FUS P525L/XCK1ε kinase domain, or FUS P525L/CK1δ [site-directed]) were detected as a doublet migrating at positions of ∼75 and 70 kDa, whereas those expressed in FUSwt (site-directed) or FUS P525L mutant single tg flies were detected predominantly as a single band migrating at ∼70 kDa (Fig. 6,*I*–*L*). Importantly, the anti-pS182 antibody exclusively labeled the ∼75 kDa band observed in double tg FUSwt (site-directed) or P525L flies expressing CK1δ, Dco, or XCK1ε (Fig. 6,*I*–*L*). Furthermore, the ∼75 kDa band was not detected in the heads of 1-day-old FUSwt (site-directed)/Dco K38R, FUSwt (site-directed)/CK1α1 (site-directed), FUS P525L/Dco K38R, or FUS P525L/CK1α1 (site-directed) double tg flies (Fig. 6,*I*–*L*). These data suggest that CK1δ, Dco, or CK1ε phosphorylated FUS in the retina of tg flies in a kinase activity–dependent manner, supporting the notion that phosphorylation of FUS by CK1δ, its *Drosophila* homolog Dco, or CK1ε ameliorated FUS-mediated neurodegeneration in the retina of *Drosophila*. We measured the relative expression levels of FUS in the heads of single or double tg flies and found that the expression levels of FUS in heads of 1-day-old FUSwt (site-directed)/CK1δ, FUSwt (site-directed)/Dco, FUSwt (site-directed)/Dco K38R, FUSwt (site-directed)/XCK1ε kinase domain, FUSwt (site-directed)/CK1δ (site-directed), or FUSwt (site-directed)/CK1α1 (site-directed) double tg flies were 56 ± 22%, 41 ± 12%, 109 ± 9%, 65 ± 29%, 59 ± 7, and 131 ± 24% of that expressing FUSwt (site-directed) single tg flies, respectively (Fig. 6, *I* and *J*). These data indicate that CK1 phosphorylation of FUS may reduce the amount of FUS proteins in the *Drosophila* retinal photoreceptor neurons.

### Coexpression of CK1δ extended the life span of FUS wt or P525L tg flies

**Figure 7:**
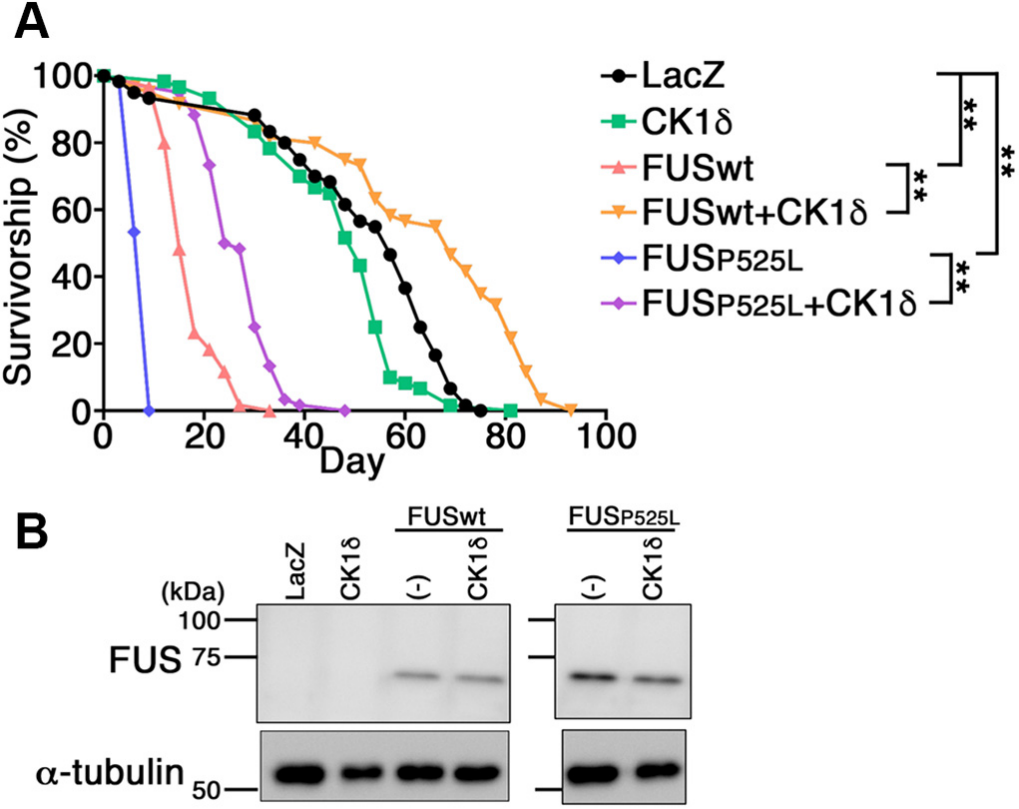
**Expression of CK1δ extended the life span of FUS wt or P525L tg flies.***A*, survival curves for LacZ (*black circles*), CK1δ (*green squares*), FUS wt (*pink triangles*), FUS wt and CK1δ (*orange inverted triangles*), FUS P525L (*blue diamonds*), or FUS P525L and CK1δ (*purple diamonds*) tg flies. Statistical analysis was performed by the log rank test, one-way ANOVA with Bonferroni post hoc test, n = 60. ∗∗*p* < 0.0001. *B*, immunoblot analyses of the heads of 1-day-old tg flies with an anti-FUS antibody (*top panel*) or an anti-α-tubulin antibody (*bottom panel*) in LacZ, CK1δ, FUS wt, FUS wt and CK1δ, FUS P525L, or FUS P525L and CK1δ tg flies. CK1, casein kinase 1; FUS, fused in sarcoma; tg, transgenic.

Finally, we aimed to examine whether phosphorylation of FUS by CK1δ affects the FUS-mediated toxicity in central nervous system neurons. To this end, we generated tg flies that singly express LacZ, CK1δ, FUS wt or FUS P525L, or doubly FUS wt and CK1δ or FUS P525L and CK1δ, in neurons under the control of *D42-GAL4* driver, which elicits expression of proteins in motor neurons and peripheral sensory neurons (47), and quantitated the survival of the flies. FUS wt and FUS P525L single tg flies exhibited significantly shortened life span compared with LacZ tg flies (Fig. 7*A*). Coexpression of CK1δ in the motor neurons of FUS wt or FUS P525L tg flies significantly extended the life span (Fig. 7*A*). Immunoblot analyses of the lysate of heads of tg flies revealed that FUS wt tg flies and FUS wt/CK1δ double tg flies expressed similar levels of FUS proteins, and that FUS P525L tg flies and FUS P525L/CK1δ double tg flies also expressed similar levels of FUS proteins (Fig. 7*B*). These data suggest that phosphorylation of FUS by CK1δ mitigates the FUS-induced toxicity in the motor neurons of *Drosophila*.

## Discussion

In this study, we have shown that CK1δ and CK1ε phosphorylated human FUS protein *in vitro*, in HEK293 cells, and in the photoreceptor neurons of *Drosophila in vivo*. Mass spectrometric analysis revealed that residues Ser163, Ser164, Ser182, Ser183, Ser221, Ser273, Ser277, Thr338, Ser346, and Ser462 are the *in vitro* phosphorylation sites of human FUS by CK1δ (Fig. 1), among which the phosphorylation of Ser182 in HEK293 cells and *Drosophila* retina was confirmed by using a phosphorylation site–specific antibody (Figs. 2, and 3). CK1 family consists of serine/threonine protein kinases phylogenetically conserved from yeast to humans. Human CK1δ and CK1ε share highly homologous amino acid sequences, 98% identical in the kinase domain and 53% in the carboxy-terminal regulatory domain, respectively (36, 48). The consensus phosphorylation sequence for the CK1 family kinases is pS/T-X-X-S/T or D/E-X-X-S/T, where pS/T denotes phosphorylated serine or threonine, D/E aspartic acid or glutamic acid, X is any amino acid, and the S/T represents the target residue, respectively (49). Among the 10 identified *in vitro* phosphorylation sites by CK1δ, Ser183 (_180_DQSS_183_), Ser273 (_270_DQGS_273_), and Ser346 (_343_DPPS_346_) fit into the consensus phosphorylation sequence noted previously, whereas other seven residues do not. Previous studies revealed that phosphoinositide 3-kinase–like kinase family kinases, for example, DNA-PK and ataxia–telangiectasia mutated, phosphorylate the S/T-Q motif in the LC domain of FUS (Fig. 1*D*) (26, 31, 32). Phosphorylation sites by DNA-PK are unlikely to overlap with those phosphorylated by CK1δ, because (i) any of the 10 phosphorylated Ser/Thr residues we identified by *in vitro* incubation with CK1δ did not fit into the S/T-Q motif and (ii) treatment with calicheamicin, an activator of DNA-PK, did not elicit the Ser182 phosphorylation of FUS in HEK293 cells, despite the retarded mobility indicative of FUS phosphorylation (Fig. 2*E*). Interestingly, seven of the 10 phosphorylated residues by CK1δ were located within or in the vicinity of the G-rich domain, that is, the carboxy half of the LC domain, whereas the phosphorylated serine/threonine residues by DNA-PK are exclusively located within the QGSY-rich domain, that is, the amino half of the LC domain. The discrete separation of the phosphorylation sites by CK1 and DNA-PK, respectively, may suggest that the tertial structure of FUS, especially that of the LC domain, determines the susceptibility of FUS by different protein kinases, thereby differentially altering the structure of FUS. We revealed that FUS was phosphorylated by CK1δ or CK1ε *in vitro*, in HEK293 or SH-SY5Y cells, and the retinal photoreceptor neurons in *Drosophila*. Coincubation of PF670462, a selective CK1δ or CK1ε inhibitor, inhibited the phosphorylation of FUS by CK1δ or CK1ε *in vitro* and in HEK293 cells (Figs. 1*B* and 2*G*, and *H*), and a kinase-dead mutant of CK1δ did not elicit FUS phosphorylation in HEK293 cells and retina of *Drosophila* (Figs. 2*F* and 6,*I* and *K*). These data collectively suggest that the kinase activity of CK1δ or CK1ε is necessary for the phosphorylation of FUS. However, we cannot rule out the possibility that other kinase(s) that were activated by CK1δ or CK1ε were directly responsible for the phosphorylation of FUS protein within cells. No anti-pS182-positive bands were detected in the HEK293 cells transfected with FUSwt or FLAG-FUSwt (Fig. 2, *A* and *C*), which may suggest that the amount of Ser182-phosphorylated FUS was below the detection limit in HEK293 cells without coexpression of CK1δ or CK1ε.

It is still unclear how phosphorylation of FUS by CK1δ affects the tertial structure of FUS protein. Crystal structure analysis of the fibrils formed from the 214-residue LC domain of FUS by the solid-state nuclear magnetic resonance revealed that residues 39 to 95 formed core structure of FUS-LC fibrils with one dynamic loop in residues 55 to 62 (26). The core region of FUS-LC fibrils also was identified as the low-complexity aromatic-rich kinked segments characterized by the kinked β-sheet structures (27). Interestingly, phosphorylation of FUS by DNA-PK reduced the hydrogel formation presumably through suppression of the hydrogen bond network among Ser84, Tyr75, and Thr78 (26). Recently, cryo-electron microscopic imaging revealed that the molecular structure of the fibrils formed from the LC domain composed of residues 111 to 214 of FUS: within the FUS fibrils, residues 112 to 150 formed the core structure adopted by the U-shaped conformation, which was stabilized by a plethora of hydrogen bonds involving side chains of Gln, Asn, Ser, and Tyr residues (50, 51). Because CK1δ phosphorylated Ser163, Ser164, Ser182, and Ser183 of FUS *in vitro*, it is conceivable that CK1δ phosphorylation of FUS interrupted the hydrogen bonds within residues 111 to 214 of FUS, which resulted in the inhibition of formation of FUS polymers. Further structural analyses of FUS, especially that of the LC domain after phosphorylation by CK1δ or CK1ε, will provide insights into the changes in the tertial structure of FUS caused by phosphorylation.

In this study, we showed that coexpression of human CK1δ significantly ameliorated the degeneration of photoreceptor neurons induced by overexpression of wt or P525L mutant FUS in the retina of tg flies (Figs. 5, and 6) and increased the life span of tg flies that express wt or P525L mutant FUS in motor and sensory neurons (Fig. 7). These results strongly support the notion that the phosphorylation of FUS by CK1δ ameliorated the FUS-mediated neurodegeneration. Although the mechanism whereby phosphorylation of FUS attenuates its toxicity remains elusive, one possibility would be that the phosphorylation directly inhibited the formation of toxic species of FUS causative to neurodegeneration. Biochemical analysis of the postmortem brains of patients with FTLD-FUS showed an increase in the level of radioimmunoprecipitation assay-insoluble FUS (42). It has been shown that fALS-linked R521C mutant FUS expressed in U87 cells was exclusively extracted in the 1% NP-40-insoluble fraction, whereas wt FUS was predominantly retrieved in the 1% NP-40-soluble fraction (52). These data altogether suggest a causal link between the increased insolubility of FUS and the pathogenic mechanism of FUS proteinopathies. Our finding that FUS phosphorylated by CK1δ or CK1ε, migrating at slower positions compared with nonphosphorylated FUS on SDS-PAGE, was exclusively fractionated into the 1% NP-40-soluble fraction (Fig. 3) may support the assumption that phosphorylation of FUS by CK1δ or CK1ε counteracted the conformational changes leading to the formation of the toxic species of FUS that acquired detergent insolubility. The relative levels of FUS proteins in the heads of FUSwt (site-directed) flies doubly expressing CK1δ, Dco, or XCK1ε kinase domain were reduced compared with those in FUSwt single tg flies (Fig. 6*I*). Thus, we cannot rule out the possibility that the reduction of FUS protein caused by phosphorylation by as yet unknown mechanism(s) that took place in the *Drosophila* retinal photoreceptor neurons might have partly contributed to the amelioration of retinal degeneration induced by FUS. However, the reduction in the levels of FUS protein was observed neither in HEK293 cells doubly transfected with FLAG-FUSwt and myc-CK1δ or myc-CK1ε (Fig. 2*A*) nor in the head of *Drosophila* doubly expressing FUSwt with CK1δ under the control of *D42-GAL4*-driver (Fig. 7*B*); this suggests that the reduction of FUS through phosphorylation by CK1δ or CK1ε is likely a cell type–dependent event. Further elucidation of the identity of the toxic FUS species, as well as of its downstream pathways, will be needed to unravel the whole picture of the FUS-mediated neurodegeneration. Previous studies revealed that phosphomimetic substitutions within the QGSY-rich domain of FUS, including residues phosphorylatable by DNA-PK, mitigated FUS-induced toxicity in yeast (33, 53). We have tested the levels of phosphorylation and the solubility of FUS mutated at either of the four phosphorylation sites by CK1δ or CK1ε, that is, Ser163, Ser164, Ser182, and Ser183, into nonphosphorylatable alanine or phosphorylation-mimic aspartate in HEK293 cells but observed neither changes in the mobility of FUS polypeptides on SDS-PAGE nor their solubility (data not shown). These results suggest that multiple phosphorylated Ser/Thr residues in the LC domain of FUS might have collectively contributed to the solubility of FUS.

FUS shares striking functional, structural, and neuropathological similarities with TDP-43, the latter being another RNA-binding protein causative to ALS/FTLD. This leads one to speculate that the pathophysiology of neurodegeneration caused by FUS and TDP-43 in ALS/FTLD may share common mechanisms (8, 9, 13). However, the effect of CK1 phosphorylation on TDP-43 and FUS may be divergent. Phosphorylation of TDP-43 by CK1δ has been shown to trigger the accumulation of TDP-43 in SH-SY5Y cells and NSC-34 motor neuron–like cells (41, 54). In the *Drosophila in vivo* models, coexpression of Dco in M337V or Q331K ALS-mutant TDP-43 tg flies promoted the formation of aggregates and enhanced the toxicity in retina (55). This contrasts with our present results that phosphorylation by CK1δ or CK1ε increased the solubility of FUS in HEK293 cells (Fig. 3), and that coexpression of human CK1δ or Dco with FUS in the photoreceptor neurons ameliorated the toxicity induced by the expression of wt or P525L ALS-mutant FUS (Figs. 5, and 6). These interesting differences in the pathophysiological function of CK1 may provide us with clues to the pathomechanism of proteinopathies caused by TDP-43 and FUS. Further investigations into the structural and functional changes in FUS protein by CK1δ/ε phosphorylation will unveil the molecular mechanisms and pave the way toward the therapeutic interventions into FUS proteinopathies, for example, through activation of CK1δ/ε.

## Experimental procedures

### Plasmid construction

For the expression of human FUS in *E. coli*, human FUS complementary DNA (cDNA) was subcloned between BamHI and XhoI sites of pGEX-6P-1 vector (GE Healthcare), and for the mammalian expression of FLAG-tagged human FUS wt or P525L, cDNA was subcloned between BamHI and XhoI sites of pcDNA5 vector (Thermo Fisher Scientific) as previously described (28), and FUS wt or P525L cDNA without epitope tag was subcloned between BamHI and XhoI sites of pcDNA3.1(+) vector (Thermo Fisher Scientific). For attB-attP40 *Drosophila* expression system, human FUS wt cDNA was subcloned between XhoI and XbaI sites of pUAS-attB vector (56). AllS mutant FUS cDNA was provided by Drs Masato Kato and Steven L. McKnight (University of Texas Southwestern Medical Center) (28). pCS2-Myc-CK1α1, pCS2-Myc-CK1δ, and pCS2-Myc-CK1ε vectors were provided by Drs Cheong Jit Kong and David M. Virshup (Duke-NUS Graduate Medical School Singapore). pcDNA3.1-FLAG-CK1δ1-317 or pcDNA3.1-FLAG-CK1δ1-317 K38R vector was previously reported (41). pUAS-attB vector was provided by Drs Tomonori Katsuyama and Masayuki Miura (The University of Tokyo).

### Antibodies

The following antibodies were used as primary antibodies, anti-FUS(400–450) (Bethyl; catalog no.: A300-293A), anti-FLAG (Sigma; catalog no.: M2), anti-myc (Cell Signaling Technology; catalog nos.: 9B11 and 71D10), anti-α-tubulin (Sigma; catalog no.: DM1A). A rabbit polyclonal anti-pS182 antibody was raised against a synthetic phosphopeptide of FUS (GNYGQDQ(pS)SMSSGGG, pS denotes phosphoserine). For a polypeptide absorption assay, 2 μl of anti-pS182 antibody was preincubated with 1.9 μg (∼1.3 nmol) of nonphosphorylated polypeptide (GNYGQDQSSMSSGGG) or 2.0 μg (∼1.3 nmol) of phosphorylated polypeptide (GNYGQDQ(pS)SMSSGGG) in the absorption buffer (50 mM Tris–HCl, 150 mM NaCl, pH = 7.6 containing 0.1% of Tween-20) at 4 °C overnight, and applied for immunoblotting.

### *In vitro* kinase assay

Recombinant GST-tagged FUS protein was produced as previously described (28). Briefly, FUS/pGEX-6P-1 cDNA was transformed into BL21 (DE3) and cultured in LB medium at 18 °C. Isopropyl β-thiogalactopyranoside was added to the medium at a final concentration of 1 mM and cultured for 12 h at 18 °C. *E. coli* was collected, sonicated in lysis buffer (50 mM Tris–HCl, pH = 8.0, 50 mM NaCl, 1 mM EDTA, and 100 μM PMSF), and lysed in lysis buffer with 1% Triton X-100 for 10 min. After centrifugation at 9000*g* for 30 min, the supernatant was mixed with 50% suspension of Glutathione Sepharose 4B beads (GE Healthcare) and incubated at 4 °C overnight. Beads were washed with 0.5% Triton X-100 in PBS, incubated in elution buffer (50 mM Tris–HCl, pH = 8.0, 16 mM reduced glutathione, and 1 mM DTT) for 10 min at 4 °C, and centrifuged at 1500*g* for 5 min. To determine the purity and protein concentration of recombinant protein, purified GST-fused FUS protein samples were separated by 10% SDS-PAGE, stained with Coomassie Brilliant Blue, and the protein concentrations were determined by the band intensities using ImageJ software (NIH). For the kinase assay, 4 μg of recombinant GST-tagged FUS protein were resuspended in 24 μl of an assay buffer (50 mM Tris–HCl, pH = 7.5, 20 mM MgCl_2_, 2 mM DTT, 100 μM EGTA, and 1 mM ATP), and incubated with 0.2 μg of recombinant human CK1δ protein (Abcam; catalog no.: ab103955) for 1 h at 30 °C. The reaction was stopped by the addition of Laemmli sample buffer and boiling. For alkaline phosphatase treatment, 2.3 μg of recombinant GST-tagged FUS protein was resuspended with 23 ng of recombinant human CK1δ protein in 14 μl of assay buffer. The mixture was incubated with 8.5 U of alkaline phosphatase (Roche) in the dephosphorylation buffer (Roche) for 1 h at 37 °C. For PF670462 treatment, 2 μg of recombinant GST-tagged FUS was resuspended with 20 ng of recombinant human CK1δ and 10 μM of PF670462 (Cayman Chemical) in 12 μl of assay buffer and incubated for 1 h at 30 °C.

### Mass spectrometric analysis

Mass spectrometric analysis was performed as previously reported (40, 41). In brief, phosphorylated FUS was separated by SDS-PAGE, and the gel was excised and soaked in 50 mM Tris–HCl (pH = 8.0) containing 50% acetonitrile for 30 min. The gel was dried in a Speed-Vac and incubated in 50 mM Tris–HCl (pH = 8.0) containing 250 ng of chymotrypsin (Roche) at 37 °C for 20 h. The digests were extracted from the gel with 100 μl of 0.1% TFA containing 60% acetonitrile, evaporated in a Speed-Vac, resuspended in 0.1% formic acid containing 2% acetonitrile, and applied to a DiNa HPLC system (KRA Technology Corp) with an automatic sampler. A packed nanocapillary column (Nikkyo Techno Co Ltd; catalog no.: NTCC-360/75-3-123) was used at a flow rate of 200 nl/min with a 2 to 80% linear gradient of acetonitrile in 0.1% formic acid. Eluted peptides were detected directly with an ion trap mass spectrometer (Velos Pro; Thermo Fisher Scientific). The obtained spectra were analyzed with Proteome Discoverer, version 1.41 (Thermo Fisher Scientific) and Mascot software, version 2.51 (Matrix Science). To identify the peptides derived from the phosphorylated FUS band, we used the mass spectrometry search parameters listed in Table S2.

### Cell cultures and transfection

HEK293 cells (American Type Culture Collection; catalog no.: CRL-1573) were cultured in Dulbecco’s modified Eagle's medium with 10% fetal bovine serum and 100 units/ml penicillin and 100 μg/ml streptomycin. SH-SY5Y cells (American Type Culture Collection; catalog no.: CRL-2266) were cultured in Dulbecco’s modified Eagle's medium/F-12 medium with 10% fetal bovine serum. Plasmid DNA was introduced into HEK293 cells or SH-SY5Y cells using FuGENE 6 Transfection Reagent (Promega) according to the manufacturer’s protocol. Cells were analyzed 24 h after transfection for immunocytochemistry and 48 h after transfection for immunoblotting. About 20 nM of calicheamicin was added to the medium 45 h after transfection. PF670462 was added to the medium 24 h after transfection.

### Immunocytochemistry

HEK293 cells or SH-SY5Y cells were fixed with 4% paraformaldehyde in PBS for 30 min at room temperature. After blocking with 10% calf serum in PBS containing 0.1% of Triton X-100, cells were incubated with primary antibodies for overnight at 4 °C. For immunofluorescence, cells were incubated with a mixture of Alexa fluorophore–conjugated secondary antibodies against mouse or rabbit immunoglobulin G and DRAQ5 for HEK293 cells or 4′,6-diamidino-2-phenylindole for SH-SY5Y as a nuclear marker. Cells were observed with FV3000 confocal microscope (Olympus) or SpinSR10 (Olympus).

### Immunoblotting

HEK293 cells, SH-SY5Y cells, or 10 heads of 1-day-old male flies were lysed in a Laemmli sample buffer with PhosSTOP phosphatase inhibitor cocktail (Roche). The lysates were separated by 7.5 or 10% SDS-PAGE and transferred to polyvinylidene difluoride membranes. After probing with primary antibodies, the immunoblots were developed using a chemiluminescence kit (Wako) or SuperSignal West Femto Chemiluminescent Substrate (Thermo Fisher Scientific), and visualized by LAS-4000 mini (GE Healthcare). The band intensities of FUS protein derived from HEK293 cells were quantified by ImageQuant (GE Healthcare). The band intensities of FUS or α-tubulin protein derived from the heads of each tg fly line were quantified by ImageQuant, and the average relative level of FUS (FUS/α-tubulin) was calculated in three or five independent experiments.

### Sequential extraction of soluble and insoluble proteins

Sequential extraction of soluble and insoluble proteins was performed as previously described (28). In brief, HEK293 cells were collected, lysed in NP-40 buffer (containing 1% NP-40, 20 mM Tris–HCl, pH = 7.4, 150 mM NaCl, 5 mM EDTA, 10% glycerol, 1 mM DTT, 10 mM sodium fluoride, 1 mM sodium orthovanadate, and 5 mM sodium pyrophosphate), and rotated for 30 min at 4 °C. After centrifugation at 20,000*g* for 15 min, the supernatant was collected as a detergent-soluble fraction. The pellet was washed with NP-40 buffer twice and sonicated in urea–SDS buffer (containing 8 M urea and 3% SDS in NP-40 buffer). After centrifugation at 20,000*g* for 15 min, the supernatant was collected as a detergent-insoluble fraction. cOmplete Protease Inhibitor Cocktail (Roche) was used to avoid protein degradation.

### Fly stocks and generation of tg flies

Tg flies expressing *UAS-FUS wt* or *P525L* were previously generated (28). The attB-attP40 expression system was used for the generation of tg flies expressing *UAS-FUSwt (site-directed)*. *gmr-GAL4*, *d42-GAL4*, *UAS-LacZ*, *UAS-Dco*, *UAS-Dco K38R*, *UAS-human CK1δ*, *UAS-Xenopus CK1ε kinase domain*, *UAS-human CK1δ (site-directed)*, *UAS-human CK1α1 (site-directed)* lines were purchased from Bloomington *Drosophila* Stock Center. Fly stocks were raised on standard *Drosophila* medium at 20 °C. Crosses between the *Drosophila* strains were carried out using standard procedures at 20 °C for the measurement of life span, 25 °C for other experiments.

### External surface observation of flies

Adult flies (20-day-old female) were anesthetized with CO_2_, and the outer surface of eyes was observed with zoom stereo microscope (Olympus SZ-PT). For the quantification of the pigmented areas of tg flies, a comparable area of eyes for each genotype was selected. Quantification of the region without pigment loss in the selected area of eyes was analyzed using the ImageJ software.

### Immunohistochemistry

Immunohistochemistry of tg flies was performed as previously described (28). Briefly, heads of 5-day-old or 10-day-old female flies were fixed with 4% paraformaldehyde in PBS containing 0.3% Triton X-100 at room temperature for 2 h. Fixed heads were embedded in paraffin and cut in coronal sections at 4 μm thickness. Hematoxylin–eosin staining was performed to evaluate retinal structures, and retinal thickness was quantified as an average of two measurements at the central area per an eye with ImageJ software. A minimum of 10 eye sections that were cut at the center of retina perpendicularly to the eye surface were measured per line. For comparison between lines, Dunnett’s test was applied.

### Measurement of life span

Life span of tg flies was measured as previously described (57). In brief, male flies 0 to 72 h after eclosion were collected into fresh food vials at a density of 20 flies per vial. The vials were kept at 25 °C. Every third day, flies were transferred to new food vials, and the number of dead flies was recorded. Three vials (60 flies) were tested per each genotype. Statistical analysis was performed by the log rank test, using Prism 6 for Mac OSX (GraphPad Software, Inc).

## Data availability

Raw mass spectrometry data are deposited in jPOST (https://repository.jpostdb.org/) (JPST001574, PXD033730). The datasets and materials used during the current study are available from the corresponding authors on reasonable request.

## Supporting information

This article contains supporting information.

## Conflict of interest

The authors declare that they have no conflicts of interest with the contents of this article.

## Figures and Tables

**S-1:**
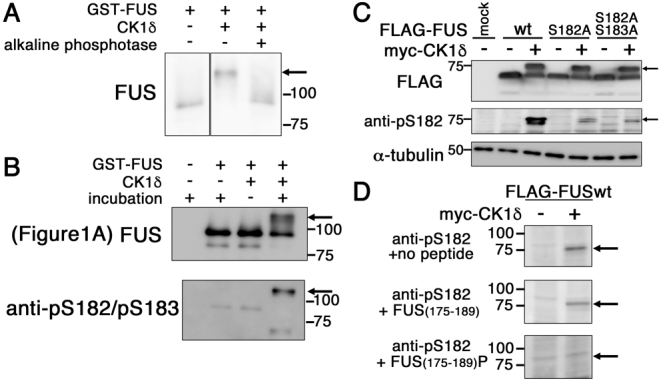


**S-2:**
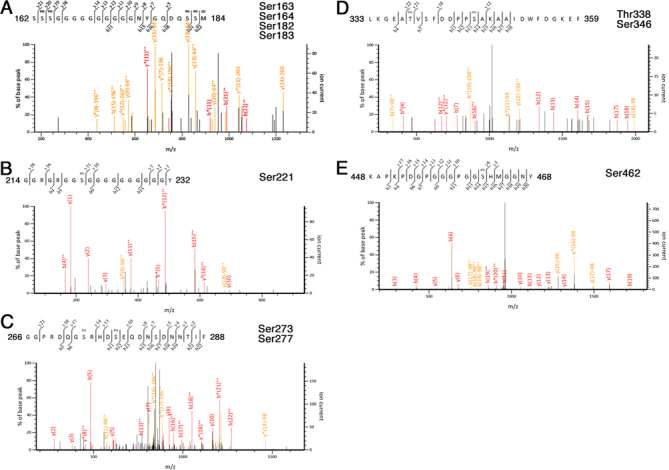


**S-3:**
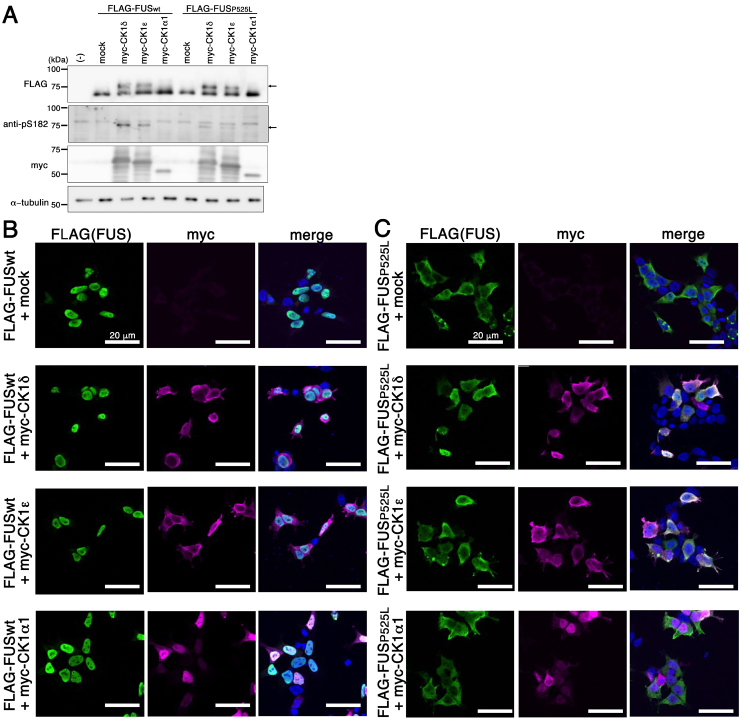


**S-4:**